# Supplementary material for: A fast and efficient colocalization algorithm for identifying shared genetic risk factors across multiple traits
Source: Nat Commun. 2021 Feb 3;12:764. doi: 10.1038/s41467-020-20885-8 (PMC7858636; doi:10.1038/s41467-020-20885-8)
Supplement: Supplementary file 3 — Reporting Summary [file 41467_2020_20885_MOESM3_ESM.pdf]

nature research

Corresponding author(s): Christopher Foley

Last updated by author(s): Nov 19, 2020

Reporting Summary

Nature Research wishes to improve the reproducibility of the work that we publish. This form provides structure for consistency and transparency in reporting. For further information on Nature Research policies, see our Editorial Policies and the Editorial Policy Checklist.

Statistics

For all statistical analyses, confirm that the following items are present in the figure legend, table legend, main text, or Methods section.

n/a	Confirmed	
☐	☒	The exact sample size (*n*) for each experimental group/condition, given as a discrete number and unit of measurement
☐	☒	A statement on whether measurements were taken from distinct samples or whether the same sample was measured repeatedly
☐	☒	The statistical test(s) used AND whether they are one- or two-sided<br>*Only common tests should be described solely by name; describe more complex techniques in the Methods section.*
☐	☒	A description of all covariates tested
☐	☒	A description of any assumptions or corrections, such as tests of normality and adjustment for multiple comparisons
☐	☒	A full description of the statistical parameters including central tendency (e.g. means) or other basic estimates (e.g. regression coefficient) AND variation (e.g. standard deviation) or associated estimates of uncertainty (e.g. confidence intervals)
☐	☒	For null hypothesis testing, the test statistic (e.g. *F*, *t*, *r*) with confidence intervals, effect sizes, degrees of freedom and *P* value noted<br>*Give P values as exact values whenever suitable.*
☐	☒	For Bayesian analysis, information on the choice of priors and Markov chain Monte Carlo settings
☐	☒	For hierarchical and complex designs, identification of the appropriate level for tests and full reporting of outcomes
☐	☒	Estimates of effect sizes (e.g. Cohen's *d*, Pearson's *r*), indicating how they were calculated

Our web collection on statistics for biologists contains articles on many of the points above.

Software and code

Policy information about availability of computer code

Data collection	SNPs marking the colocalization signal shared across two or more traits and their annotation in the Ensembl Variant Effect Predictor were obtained from PhenoScanner (http://www.phenoscanner.medschl.cam.ac.uk).
Data analysis	We developed (and made publicly available) an R package for performing the HyPrColoc analyses (Version: v1.0.0; https://github.com/jrs95/hyprcoloc and https://github.com/cnfoley/hyprcoloc). The regional association plots (as seen in Figure 8) were created using gassocplot (https://github.com/jrs95/gassocplot) and LD information from 1000 Genomes (http://www.internationalgenome.org/). We compared the performance of HyPrColoc with the publicly available software packages: COLOC (Version: 3.2-1; https://cran.r-project.org/web/packages/coloc/); eCAVIAR (Version: 2.0.0; https://github.com/fhormoz/caviar); and MOLOC (Version: 0.1.0; https://github.com/clagiamba/moloc).

For manuscripts utilizing custom algorithms or software that are central to the research but not yet described in published literature, software must be made available to editors and reviewers. We strongly encourage code deposition in a community repository (e.g. GitHub). See the Nature Research guidelines for submitting code & software for further information.

Data

Policy information about availability of data

All manuscripts must include a data availability statement. This statement should provide the following information, where applicable:

- Accession codes, unique identifiers, or web links for publicly available datasets
- A list of figures that have associated raw data
- A description of any restrictions on data availability

The genome-wide association summary data that support the findings of this study are available from: CARDIoGRAMplusC4D (http://www.cardiogramplusc4d.org) for coronary heart disease; MAGIC (www.magicinvestigators.org) for glycaemic traits; GLGC (www.lipidgenetics.org) for lipid measures; TAG (https://www.med.unc.edu/pgc/results-and-downloads) for smoking; SSAGC (www.thessgac.org) for years in education; DIAGRAM (https://www.diagram-consortium.org)

for type 2 diabetes; CKDGen (http://ckdgen.imbi.uni-freiburg.de) for renal function measure eGFR; Okada et al. (plaza.umin.ac.jp/~yokada/datasource/software.htm) for rheumatoid arthritis; and the first release of the Neale Lab's GWAS analysis of UK-Biobank (http://www.nealelab.is/uk-biobank) for the adiposity measures and blood pressure traits. The summary data on gene expression and protein expression in whole blood are available from eQTLGen (http://www.eqtlgen.org/cis-eqtls.html) and Sun et al. (https://www.phpc.cam.ac.uk/ceu/proteins/), respectively. The LD information was computed using the phased haplotypes from the 1000 Genomes study (http://www.internationalgenome.org/). Full results from the genome-wide colocalization analysis of CHD and 14 related traits using HyPrColoc are available at https://jrs95.shinyapps.io/hyprcoloc_chd.

Field-specific reporting

Please select the one below that is the best fit for your research. If you are not sure, read the appropriate sections before making your selection.

☒ Life sciences ☐ Behavioural & social sciences ☐ Ecological, evolutionary & environmental sciences

For a reference copy of the document with all sections, see nature.com/documents/nr-reporting-summary-flat.pdf

Life sciences study design

All studies must disclose on these points even when the disclosure is negative.

Sample size	We assessed performance of each method over a range of study samples sizes - selected to broadly reflect the variety of sample sizes considered in the original COLOC study (Giambartolomei C et al. (2014) Bayesian Test for Colocalisation between Pairs of Genetic Association Studies Using Summary Statistics. PLoS Genet 10(5): e1004383.) as well as the subsequent analyses of Wallace (Wallace C (2020) Eliciting priors and relaxing the single causal variant assumption in colocalisation analyses. PLOS Genetics 16(4): e1008720.)
Data exclusions	No data were excluded.
Replication	In the assessment of coronary heart disease, we evaluated the quality of our findings by quantifying the proportion of genomic loci and putative causal variants identified by HyPrColoc which matched genomic loci and variants known to be associated with coronary heart disease. 88% of the genomic loci identified by HyPrColoc replicated either known candidate causal SNPs and/or the nearest gene.
Randomization	This is not relevant. Our study uses summary level data, i.e. regression coefficients and standard errors, from genomewide association studies.
Blinding	This is not relevant. Our study uses summary level data, i.e. regression coefficients and standard errors, taken from genomewide association studies.

Reporting for specific materials, systems and methods

We require information from authors about some types of materials, experimental systems and methods used in many studies. Here, indicate whether each material, system or method listed is relevant to your study. If you are not sure if a list item applies to your research, read the appropriate section before selecting a response.

Materials & experimental systems

n/a	Involved in the study	
☒	☐	Antibodies
☒	☐	Eukaryotic cell lines
☒	☐	Palaeontology and archaeology
☒	☐	Animals and other organisms
☒	☐	Human research participants
☒	☐	Clinical data
☒	☐	Dual use research of concern

Methods

n/a	Involved in the study	
☒	☐	ChIP-seq
☒	☐	Flow cytometry
☒	☐	MRI-based neuroimaging
